# The neuro-immune crosstalk between periphery and central nervous system during acute immune response to virus-mimicking RNA in parrots

**DOI:** 10.1098/rsos.251343

**Published:** 2025-10-08

**Authors:** Balraj Melepat, Daniel Divín, Kateřina Marková, Tao Li, Nithya Kuttiyarthu Veetil, Eleni Voukali, Lucie Schmiedová, Martin Těšický, Michal Vinkler

**Affiliations:** ^1^ Department of Zoology, Faculty of Science, Charles University, Praha, Czech Republic; ^2^ Institute of Vertebrate Biology, Czech Academy of Sciences, Brno, Jihomoravský, Czech Republic; ^3^ Faculty of Veterinary Medicine, Ludwig Maximilian University, München, Bayern, Germany

**Keywords:** avian immunology, cytokine signalling, gastrointestinal tract, neuro-immune regulation, neuroimmunology, neural inflammation

## Abstract

Parrots, valued companion animals with a concerning conservation status, can act as reservoirs for zoonotic diseases. During various infections, systemic inflammation significantly impairs host health. However, the regulation of inflammatory responses in birds, particularly in cognitively advanced parrots, remains poorly understood. Here, we examine parrot systemic inflammation in response to virus-mimicking stimulation. In budgerigars (*Melopsittacus undulatus*), a novel model for neuroinflammation research, we induced sterile inflammation with synthetic poly(I:C) RNA and analysed dose-, time- and tissue-dependent gene expression patterns of key markers, including *TLR3*, *NLRP3*, *CASP1*, *IL1B* and *IL6,* during acute immune response. We report a significant relationship between cytokine expression (*IL1B*, *IL6*) in the intestine (local response) and brain (systemic response) that has not yet been described after viral stimulation in parrots. Peripheral *IL6* expression was upregulated at 3–6 h after stimulation with poly(I:C). In the brain, multiple genes (*TLR3*, *IL1B* and *IL6*) showed activation early during the immune response. These findings highlight the susceptibility of parrots to neuroinflammation following viral infections, having specific relevance for basic research in neurobiology, immunology and behavioural science, and also veterinary research in psittacine birds. Our study provides a foundation for future comparative research on avian neuro-immune crosstalk and neuroinflammation-related behavioural disorders.

## 1. Introduction

Parrots (Psittaciformes) represent the most popular companion birds kept by humans [1]. Current estimates suggest that about half of their global population is domestic [2]. Viral infections represent a serious threat to humans and parrots [3–5]. Because birds are involved in the transmission of 18.4% of emerging and zoonotic diseases [3], understanding psittacine health is highly relevant. Parrots frequently transmit psittacosis or influenza [6–8]. Captive parrots frequently suffer from various health issues, including digestive and behavioural disorders [9,10]. Recent research suggests that some of these disorders may be immune-mediated [11]. Several viral diseases that are common in parrots, such as Newcastle disease and Borna viruses, cause severe neurological disorders in birds [4,12] and, notably, the Borna viruses are also suspected to pose a serious risk to humans [13]. Despite these risks, our understanding of interspecific variation in avian immune function remains limited in comparison to mammals [14]. Most current insights are derived from studies in the domestic chicken, the primary avian model species [15]. However, the substantial phylogenetic and ecological diversity among birds points to significant heterogeneity in their immune defence mechanisms [16–19]. Thus, advancing our knowledge of immune regulation in parrots could not only enhance their veterinary care but also establish them as a valuable model taxon in comparative immunology. Our earlier research suggested that parrots may be particularly valuable for research in neuroimmunology. Because of their genomic loss of the *CNR2* gene, which regulates the neuro-immune interplay, they may be naturally susceptible to disorders associated with neuroinflammation [11].

Inflammation is a complex biological process that is linked to collateral self-damage [20]. The equilibrium between pathogen clearance and self-damage becomes particularly crucial when inflammation affects the central nervous system (CNS), where neurons generally lack regenerative capacities [21]. Therefore, inflammation requires precise regulation, which is mediated by cytokines [22–24]. Assessing the expression levels of the pro-inflammatory and anti-inflammatory cytokines offers valuable insight into the dynamics of inflammation regulation in both peripheral and central nervous tissues [22,23]. Similar to bacterial infections, viral pathogens also typically trigger tissue-specific responses in the periphery [25,26]. Avian immunity detects pathogens through pattern recognition receptors (PRRs), including toll-like receptors (TLRs) or NOD-like receptors (NLRs) [27,28]. Among these, toll-like receptor 3 (TLR3) and the NLR family pyrin domain-containing protein 3 (NLRP3) are well known for canonically activating inflammation during viral infections [29–31]. In birds, both viral double-stranded RNA and synthetic polyinosinic:polycytidylic acid [poly(I:C)] are recognized by TLR3, which induces upregulation of pro-inflammatory cytokines such as interleukin 1β (IL1B) or interleukin 6 (IL6), both *in vivo* and *in vitro* [27,30]. A substantial increase in IL1B and IL6 in the periphery can lead to the activation of brain glial cells, resulting in pathological neuroinflammation [32–34]. Poly(I:C) has previously been applied in the periphery to induce neuroinflammation in both mammals and chickens [35–38]. Given their possibly impaired negative regulation of neuro-immune interactions [11], this response can be particularly pronounced in parrots. In mammals (namely rodents), the immune response affecting neural regulation frequently induces physiological changes including fever, sickness behaviour and anorexia [35,39,40]. Few studies targeted this response in birds [41–44].

In this study, we assessed the differential gene expression of key molecular markers of virus-induced inflammation in parrots. We propose the budgerigar (*Melopsittacus undulatus*) as a novel parrot model for investigating neuroinflammation and the neuro-immune crosstalk between the periphery and CNS [11]. In budgerigars, we triggered sterile inflammation with synthetic poly(I:C) RNA, a TLR3 ligand that mimics the viral double-stranded RNA. We followed the dose- and time-dependent patterns of this stimulation on gene expression changes in the receptors *TLR3* and *NLRP3*, the signal mediator caspase 1 (*CASP1*) and the cytokines *IL1B* and *IL6* during acute inflammatory response in the gastrointestinal tract (ileum located near the abdominal site of the peripheral stimulation) and brain (hyperpallial region in CNS affected by a systemic immune response). Our study is, to our knowledge, the first to analyse the neuro-immune crosstalk following virus-mimicking stimulation in parrots. Thus, we set a basis essential for future comparative research on neuro-immune crosstalk in birds. The research is also important for future veterinary applications in psittacine species. Given the global decline in parrot populations [45], biomedical research supporting effective conservation efforts is needed.

## 2. Material and methods

### 2.1. Experimental design

The experimental procedures mostly followed our previous experimental strategy applied for the investigation of passerine [33] and parrot [11,46,47] immune responses to bacterial lipopolysaccharide (LPS) and the previously published research on the immune response to poly(I:C) in passerine birds and rodents [42,44,48]. Briefly, 27 budgerigars:(18 females, nine males) purchased from Vyškov Zoo and from local hobby breeders (January 2022; for details see the electronic supplementary material, table S1) were transported into the animal breeding facility of the Charles University, Faculty of Science, Czech Republic, European Union. For each bird, the body weight and tarsus length were measured. The birds were marked with coloured aluminium rings bearing identification numbers and housed in cages, two per a standard 100 × 50 × 40 cm cage under regular light conditions (12 L : 12 D cycle with 1 h gradual shading, at 22°C), with ad libitum access to food and water. We allowed the birds four weeks of acclimatization before any experimental procedures. For the experiment, the 27 birds were divided into three-time groups (immune response measured after 3, 6 and 24 h), each group containing nine individuals of which three were administered with low dose of poly(I:C), three a high dose of poly(I:C) and three served as controls. The maximum stimulation period to measure the immune response was set to 24 h, which is the time at which previous research in mice reported a return of the immune activity back to its baseline [48].

**Figure 1.**
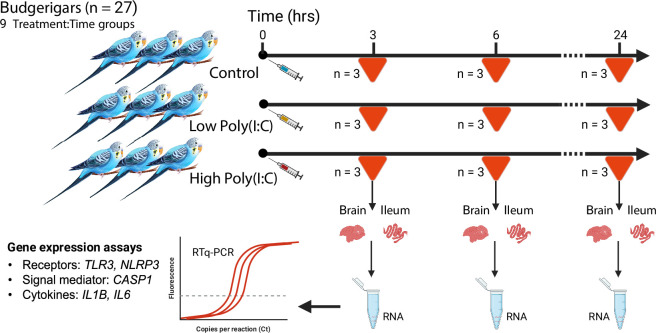
Schematic representation of the experimental design. The experimental budgerigar individuals (*n* = 27) were divided into three treatment groups; each further subdivided into three time-point categories (*n* = 3 per category). Tissue samples were collected and used for reverse transcription-quantitative polymerase chain reaction (RT-qPCR) analysis of messenger RNA expression of the target genes: *TLR3,* Toll-like receptor 3; *NLRP3,* NLR family pyrin domain-containing protein 3; *CASP1,* caspase 1; *IL1B,* interleukin 1β; *IL6*, interleukin 6. The image was created in the BioRender software (https://BioRender.com/jp24xnq) and modified.

The poly(I:C) solution used for immune stimulation in this experiment was prepared following the procedures reported in previous studies [42,48]. Briefly, 1 mg poly(I:C) (product. no. P1530, Sigma-Aldrich, MA, USA) was diluted in 100 μl of 0.9% sterile NaCl saline solution (cat. no. 200608, Unolab Manufacturing, S.L, Madrid, Spain) and was heated for 10 min to 50°C, after which it was cooled down to room temperature to achieve re-annealing. During the experimental treatment, all the individuals administered the low-dose of poly(I:C) received an intra-abdominal injection of 0.5 mg of poly(I:C) dissolved in 200 μl of sterile saline solution (approx. 12.5 mg kg^−1^). All individuals administered with high-dose poly(I:C) received 2 mg of poly(I:C) dissolved in 200 μl of sterile saline solution (approx. 50 mg kg^−1^), and the control birds were injected with 200 μl of the pure 0.9% saline solution. The dosages adopted in this experiment were selected based on the previously reported *in vivo* experiments with poly(I:C) in passerine birds [42,44]. The experiment was conducted over two consecutive days. Based on their time groups, the birds were euthanized at the time intervals of 3, 6 and 24 h. According to approved guidelines, the termination was conducted by gradual increase of the inhaled CO_2_, followed by decapitation. After the post-mortem blood collection from carotids, blood smears were prepared, and selected tissues were immediately collected (including the brain and ileum analysed in this study) and placed into the RNA-later solution where they were stored at 4°C overnight and then frozen at −80°C until analysis (details on the materials are provided in the electronic supplementary material, table S2). The blood smears collected were employed for the haematological assessment of the differential leucocyte count (for details on the procedure, see [49]). The research was approved by the Ethical Committee of the Charles University, Faculty of Science (permits13882/2011-30) and was carried out in accordance with the current laws of the Czech Republic and the European Union. A depiction of the experimental design is provided in figure 1.

### 2.2. RNA extraction and reverse transcription-quantitative polymerase chain reaction

The brain and ileum samples from all experimental individuals were homogenized in MagNa Lyser (Roche, Basel, Switzerland) using PCR clean beaded tubes (OMNI International, Kennesaw, GA, USA—cat. no.: 2150600). Subsequently, total RNA was extracted from these homogenized samples using the High Pure RNA Tissue Kit (Roche), and the quality and quantity of RNA were measured using a Nanodrop instrument (NanoDrop ND−1000) (electronic supplementary material, table S2).

Consistent with our previous research [11], gene-specific RNA expression was quantified using one-step, probe-based reverse transcription-quantitative polymerase chain reaction (RT-qPCR). While for *IL1B* and *IL6* the RT-qPCR primers, probes and synthetic complementary DNA (cDNA) standards were available from our previous experiments [46], those for *TLR3, NLRP3* and *CASP1* genes were specifically designed in this study. The new primers, probes and synthetic cDNA standards were designed using the Geneious software (version 11.1.5, Biomatters), targeting conserved regions in the avian interspecific alignments (created based on publicly available gene-specific sequences from the Ensembl database; electronic supplementary material, table S3). For coding regions covering the RT-qPCR targets within the *TLR3*, *NLRP3* and *CASP1* genes, we first designed PCR primers allowing specific amplification of a broader DNA fragment. These fragments, PCR-amplified from cDNA, were Sanger-sequenced and checked for polymorphisms that could impair RT-qPCR accuracy. The sequences were submitted to NCBI GenBank under the accession numbers OR825009-OR825034 and OR940510-OR940516. The final RT-qPCR primers, probes, and synthetic cDNA standards (gBlocks; IDT, Coralville, IA, USA) for our target genes were designed based on this input to specifically match the invariant regions of the genes (electronic supplementary material, tables S4 and S5).

For RT-qPCR, we used RNA diluted in molecular grade water supplemented with carrier tRNA (Qiagen, cat. no. 1068337) at a ratio of 1 : 5 for the target genes and 1 : 500 for the *28S rRNA* gene, which served as the reference gene. The efficiency of each primer pair set was determined from calibration curves obtained using the synthetic cDNA standards across dilution series ranging from 10^8^ to 10^2^ copies µl^−1^ [50]. The RNA samples were amplified using the Luna Universal Probe One-Step RT-PCR Kit (New England Biolabs, MA, USA—cat. no. E3006X) (electronic supplementary material, tables S2 and S6) on a Light Cycler 480 Instrument (Roche Diagnostics, Rotkreuz, Switzerland) under the conditions reported in the electronic supplementary material, table S7. All runs included template-free negative controls and freshly prepared synthetic cDNA (standard), which served as positive controls. Crossing point (Cp) values were determined by the second derivative maximum, together with the efficiency *E* values calculated using the inbuilt LightCycler480 software v.1.5.1. The gene expression quantification was calculated as standard gene expression quantity (Qst) [50], allowing comparisons of gene expression between treatments and controls (electronic supplementary material, table S8).

### 2.3. Statistical analysis

Statistical analysis was conducted using R version 4.1.0 and RStudio software version (v.2021.09.0) [51,52]. Data normality was assessed using the Shapiro–Wilk test. Given the non-Gaussian distribution of the Qst values, these were log-transformed (logQst). The effects of experimental treatment on gene expression were evaluated through testing linear models in the ‘lme4’ package, using gene expression (continuous) as the response variable and treatment, time, sex, mass and the interaction between treatment and time as explanatory variables. The minimum adequate models, defined as those in which all terms were significant at *p* ≤ 0.05, were obtained by backward elimination of non-significant terms from the full models. The backward elimination of non-significant terms was done based on the Akaike information criterion and was confirmed by changes in deviance and degrees of freedom using analysis of variance (ANOVA) with *F* statistics. Plots were generated in the ggplot2 package. The post-hoc test for the gene expression pattern and haematological parameters was performed as the Tukey HSD test. The correlation between the gene expression in the ileum and brain was checked using Pearson’s product–moment correlation tests. The correlation matrix was visualized using the corrplot package (version:0.92).

## 3. Results

### 3.1. Haematological assessment of health

To check the health status of all individuals, we performed a haematological assessment of all the experimental birds, focusing on the heterophil : lymphocyte (*H*/*L*) ratio and the relative basophil count. Although our analysis revealed some differences between the low poly(I:C) treatment group and other treatments prior to poly(I:C) stimulation (*p* = 0.030; electronic supplementary material, table S9), this was driven by a high initial *H*/*L* ratio in only two otherwise visually healthy individuals (electronic supplementary material, table S9 and figure S1). Given the relatively small sample size, we did not exclude these birds from the analysis but adjusted the interpretation of our results accordingly.

### 3.2. Differences in the expression of selected inflammation-related genes among tissues

**Figure 2.**
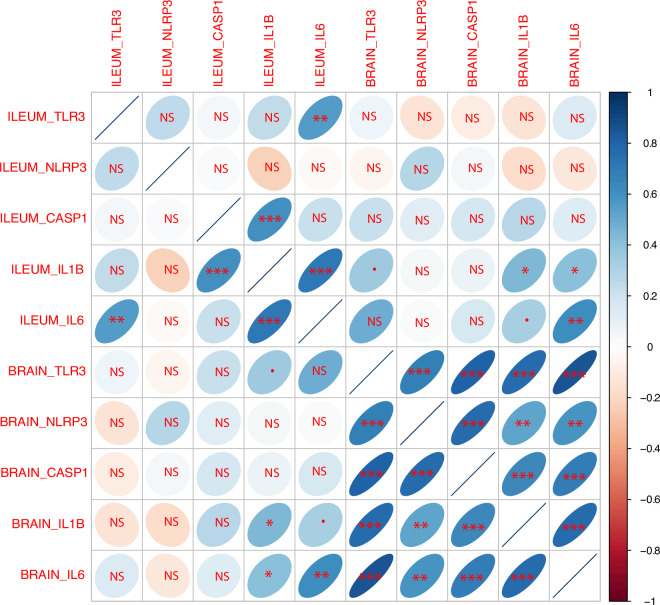
Correlation matrix comparing the relative expression of immune-related genes in the ileum and the brain during the response to poly(I:C) in the budgerigar, *n* = 27. This figure indicates joint patterns of upregulation across different members of the signalling pathway. The gene-pairs with positive correlation are depicted with positive slopes and a blue colour, and genes with negative correlation are depicted with negative slopes and a red colour. The intensity of the colour and cloud shape indicates the value of Pearson’s correlation coefficient. Statistical significance is indicated as follows: NS, non-significant;• = 0.050 < *p* < 0.100; * = 0.010 < *p* <0.050; ** = 0.010 < *p* <0.001; *** = *p* << 0.001.

We found significant correlations between the expression of different inflammation-related genes in the ileum (peripheral site induced by local stimulation) and brain (CNS site stimulated through systemic effects; figure 2; electronic supplementary material, table S10). In both tissues, *IL1B* was positively correlated with *IL6* and *CASP1* expression (in all cases *p* << 0.001). The systemic effect indicated by the association of the gene expression between the periphery and brain was observed for *IL1B* (intestinal expression linked to brain levels of *IL1B*, *p* = 0.020, and *IL6*, *p* = 0.030) as well as for *IL6* (intestinal expression linked to brain levels of *IL1B*, *p* = 0.094 and *IL6*, *p* = 0.001). Intestinal *TLR3* levels were correlated only with intestinal *IL6* (*p* = 0.003); the expression of *TLR3* in the brain was related to peripheral *IL6* (*p* = 0.011) and non-significantly also to *IL1B* (*p* = 0.066). Brain *TLR3* expression was related to *IL1B*, *IL6* and *CASP1* (in all cases *p* << 0.001). Although we found no associations between *NLRP3* expression and any other gene in the periphery, the brain *NLRP3* expression was correlated with brain levels of *IL1B* (*p* = 0.005), *IL6* (*p* = 0.001), *CASP1* (*p* << 0.001) and also *TLR3* (*p* << 0.001).

### 3.3. Intestinal expression of the selected inflammation-related genes

At the site of the local peripheral inflammation, in the ileum, *TLR3* gene expression showed no significant changes during the course of the response (full model 1 in the electronic supplementary material, table S11 and figure S2). Similarly, we observed no significant upregulation in the expression of *NLRP3* or *CASP1* in birds treated with poly(I:C) (full models 2 and 3 in the electronic supplementary material, table S11, figures S3 and S4).

**Figure 3.**
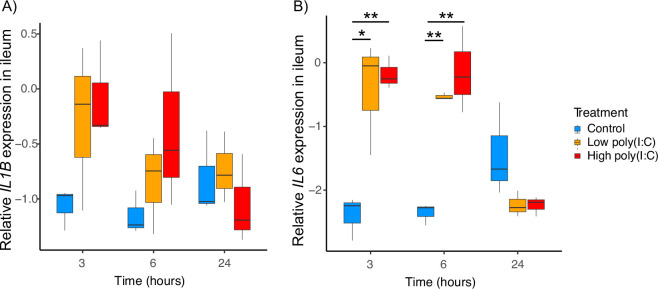
Changes in relative *IL1B* (A) and *IL6* (B) gene expression in the budgerigar ileum at different time points during the response to poly(I:C), *n* = 27. We observed upregulation in the expression of *IL1B* (trend) and *IL6* (significant difference) 3−6 h after stimulation. The cytokine gene expression is shown as logQst values on the *y*-axis, and three sampling time points (3, 5 and 24 h) are plotted on the *x*-axis. Controls (blue), low dose of poly(I:C) (orange), high dose of poly(I:C) (red). The asterisks indicate the significant differences revealed by the Tukey HSD test: * for 0.010 < *p* < 0.050, ** for 0.001 < *p* < 0.010 (for details see the electronic supplementary material, table S12).

For the *IL1B* gene, the full model analysis indicated a significant upregulation in the gene expression only at 3 h after stimulation with the high poly(I:C) dose (*p* = 0.043; full model 4 in the electronic supplementary material, table S11; and figure 3A). However, the minimum adequate model (MAM) did not support the significance of this change. Yet, we found a highly significant upregulation of *IL6* expression at 3 and 6 h after stimulation in both high-dose poly(I:C)—(Tukey HSD: 3 h, *p* = 0.008 and 6 h, *p* = 0.001, respectively; electronic supplementary material, table S12) and low-dose poly(I:C)—(Tukey HSD: 3 h, *p* = 0.013 and 6 h, *p* = 0.003, respectively; electronic supplementary material, table S12) treated birds compared to controls (MAM5: interaction treatment: time, *p* << 0.001; table 1; full model 5 in the electronic supplementary material, table S11; and figure 3B). At 24-h post-stimulation, the difference between the treatment groups and controls became non-significant.

**Table 1.** Statistically significant minimum adequate models (MAMs) for inflammation-related genes expressed during the response to poly(I:C) in the budgerigar ileum and brain*, n* = 27. (d.f., degrees of freedom. For all genes, the expression has been expressed as log(Qst) values. MAM1−4 and MAM7−8 are not listed because they were not significant.)

	tissue	MAM/variables	d.f.	*F*	*p*
MAM5	ileum	log IL6 ~ treatment + time + treatment : time	8/18	13.032	<<0.001
		treatment	6/18	13.349	<<0.001
		time	6/18	11.923	<<0.001
		treatment : time	4/18	11.844	<<0.001
MAM6	brain	log TLR3 ~ treatment	2/24	3.366	0.051
MAM9	brain	log IL1B ~ treatment + time + treatment : time	8/18	4.742	0.003
		treatment	6/18	5.962	0.001
		time	6/18	2.589	0.055
		treatment : time	4/18	3.344	0.033
MAM10	brain	log IL6 ~ treatment	2/24	10.46	<<0.001

### 3.4. Brain expression of the selected inflammation-related genes

**Figure 4.**
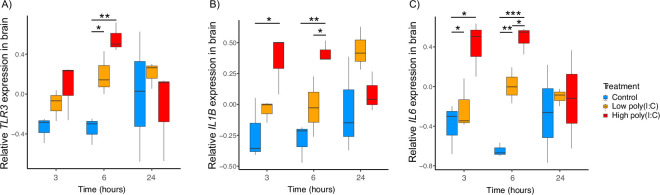
Changes in relative *TLR3* (A), *IL1B* (B), and *IL6* (C) gene expression in the budgerigar brain at different time points during the response to poly(I:C), *n* = 27. We report upregulation in the expression of *TLR3*, *IL1B* and *IL6* 3−6 h after stimulation. The *TLR3* gene expression is shown as logQst values on the *y*-axis, and three sampling time points (3, 6 and 24 h are plotted on the *x*-axis. Controls (blue), low dose of poly(I:C) (orange), high dose of poly(I:C) (red). The asterisks indicate the significant differences revealed by the Tukey HSD test: * for 0.010 < *p* < 0.050; ** for 0.001 < *p* < 0.010; *** for *p* << 0.001 (for details see the electronic supplementary material, table S12).

In contrast to the ileum, our analysis revealed a significant peak in *TLR3* gene expression in the brain at 6 h after stimulation for the high (Tukey HSD: *p* = 0.002) as well as the low poly(I:C)–dose groups compared to the controls (electronic supplementary matrial, table S12). Despite the full MAM being marginally non-significant (MAM6 : treatment; *p* = 0.051, table 1; full model 6 in the electronic supplementary material, table S11; figure 4A), this suggests a systemic PRR response to the poly(I:C) stimulation. Yet, even in the brain, we did not detect any significant changes in the expression of the *NLRP3* or *CASP1* genes (full models 7 and 8 in the electronic supplementary material, table S11, figures S5 and S6).

By contrast, for both the pro-inflammatory cytokines, our study identified highly significant time patterns of expression changes in the brain. The *IL1B* gene expression exhibited a significant upregulation in the birds treated with the high poly(I:C) dose (MAM9 : interaction treatment : time *p* = 0.033; table 1; full model 9 in the electronic supplementary material, table S11; figure 4B). The *IL1B* response to the high poly(I:C) dose started at 3 h (Tukey HSD: *p* = 0.029) and subsequently increased at 6 h (Tukey HSD: *p* = 0.006) and decreased to a non-significant difference by 24 h compared to controls (Tukey HSD: *p* = 0.818; electronic supplementary material, table S12). We also observed a very similar pattern of upregulation in *IL6,* where both high and low poly(I:C) doses triggered a significant gene expression upregulation (MAM10: treatment *p* << 0.001, table 1; full model 10 in the electronic supplementary material, table S11; figure 4C). For the high poly(I:C) treatment, the *IL6* gene expression was significantly increased at 3 h after stimulation (Tukey HSD: *p* = 0.022), reached at maximum at 6 h (Tukey HSD: *p* << 0.001), and returned to the original levels by 24 h (Tukey HSD: *p* = 0.902; electronic supplementary material, table S12). A similar pattern was observed for the low poly(I:C) dose group, but only more weakly. At 3 h after stimulation, we found no significant difference in the *IL6* gene expression compared to controls, but at 6 h, there was a significant peak in the response (Tukey HSD: *p* = 0.003), returning to the baseline at 24 h after stimulation (electronic supplementary material, table S12).

## 4. Discussion

While immune strategies are predicted to vary among species [53], knowledge of the diversity of avian immune regulation outside poultry remains limited. Our study addresses this gap by examining anti-viral immune responses in parrots, a group lacking the neuro-immune modulator CNR2 [11]. In budgerigars stimulated with poly(I:C), we found significant positive links between the expression of inflammation-related cytokines across the peripheral (ileum) and brain tissues. Surprisingly, in the periphery where the response was stimulated, we did not find any change in the expression of the receptor recognizing poly(I:C), i.e. *TLR3*. In the ileum, we detected significant upregulation of mRNA expression only in *IL6*, peaking between 3 and 6 hours after stimulation. By contrast, a complex immune response was revealed in the brain, where 3−6 h after the peripheral poly(I:C) stimulation, the *TLR3*, *IL1B* and *IL6* genes increased their expression. This indicates that the local activation of the immune response in the periphery induces a systemic response in parrots, during which neuroinflammation is triggered.

Our results show positive correlations between the intestinal and brain expression in the cytokines *IL1B, IL6* and in *CASP1*. This is similar to the findings in some other avian studies, showing consistency in expression patterns of different pro-inflammatory cytokines [50]. In the budgerigar, our findings indicate that *IL1B* and *IL6* even show correlations between the periphery and CNS, which is in line with their anticipated roles in the modulation of neuroinflammation from the periphery [54]. Interestingly, in the brain, the *IL1B, IL6* and *CASP1* levels were positively correlated with the expression of the receptors involved in poly(I:C) recognition, *TLR3* and *NLRP3*. This finding documents the complex activation of psittacine neuroinflammation, setting targets for future manipulative experiments.

Further, we questioned the time dynamics of peripheral inflammation in parrots. The selected timescale follows previous research on acute inflammation in mice treated with poly(I:C) by Cunningham *et al.* [48], who showed that during the response to poly(I:C) pro-inflammatory cytokines IL1B and IL6 peak in blood plasma at 3 h post-treatment and return to baseline levels by 24 h. Our previous study analysing parrot responses to subcutaneous injections of LPS also showed a systemic inflammatory response, with cytokine expression peaking at 6 h after stimulation [47], which is consistent with the peak *IL6* expression in response to the poly(I:C) treatment. Both poly(I:C) doses applied in our study showed similar patterns of pro-inflammatory cytokine activation, which is consistent with the results of Cunningham *et al.* [48]. Hence, our results support the view that multiple different stimuli may activate resembling patterns of neuroinflammation across species.

Brain cells such as astrocytes and microglia in humans and mice express TLR3 capable of recognizing poly(I:C) [55–58]. In our study, we found significant upregulation of *TLR3* gene expression in the brain. This is consistent with previous reports in mice, where intraperitoneal poly(I:C) treatment induced upregulation of *TLR3* gene expression in the brain 6 h after application [59]. Also, *in vitro* analyses of astrocytes treated with poly(I:C) showed upregulation of the TLR3 protein in both humans [55] and rodents [57]. In the budgerigars, *IL6* was found to be upregulated in both the ileum and brain between 3 and 6 h after stimulation with poly(I:C), suggesting that either the poly(I:C) itself or the cytokines passed the blood-brain barrier, similar to the cases previously reported in mice [59,60]. In contrast to the mouse *in vitro* and *in vivo* models [61,62], poly(I:C) treatment in parrots does not lead to any decrease in IL6 expression, which in mammals serves as a mechanism of neuronal protection. Moreover, in our study, the poly(I:C) treatment also increased the *IL1B* expression in the brain. This is consistent with our previous research on the parrot immune response to LPS [46]. These results support the hypothesis that parrots may be highly susceptible to neuronal inflammation, probably owing to the absence of CNR2, a modulator of neuro-immune interplay [11].

Previous *in vitro* research in poly(I:C)-treated mice showed that the IL1B upregulation is dependent on the NLRP3-mediated inflammatory pathway that leads to the activation of CASP1, independent of the TLR3 pathway [63,64]. In birds, *IL1B* activation is also driven through the NLRP3 pathway [65]. In a recent study conducted in chickens, Ogaili *et al.* [66] examined the expression of the *NLRP3* gene in different chicken tissues and found that LPS alone can stimulate *NLRP3* gene expression in chicken intestinal tissues. This suggests an activation mechanism slightly different from that in mammals, where LPS or poly(I:C) activate the *NLRP3* gene expression only in combination with extracellular ATP, typically associated with cell damage [64,67,68]. In rodents stimulated with poly(I:C), similar to chickens treated with LPS, the *NLRP3* expression peaks between 12 and 24 h post-stimulation [63,64]. To our knowledge, there is presently no study examining the effects of the poly(I:C) treatment on the *NLRP3* gene expression in birds. In our study, we assessed *NLRP3* expression in both the ileum and brain. However, unlike LPS-treated chickens [66], parrots did not show any significant changes in *NLRP3* expression in either tissue, even 24 h after stimulation with poly(I:C). Nevertheless, in the brain we observed a non-significant trend towards increased *NLRP3* expression in the high-dose treatments (electronic supplementary material, figure S5), which may explain the correlation between *NLRP3* and *IL1B* expression levels detected in the brain, but not in peripheral tissue. These results further support evidence for interspecific and tissue-specific heterogeneity in the regulation of inflammation across vertebrates.

Our research provides initial insights into the neuro-immune regulation directing the signalling from the periphery to the brain in parrots. Yet, the results suffer from several limitations. First, we focussed on a limited set of target genes that are key regulators of the signalling pathway linking peripheral inflammation with neuroinflammation. Unlike transcriptomic approaches that provide a broad overview of highly expressed genes [33], the RT-qPCR method employed in our study offers greater sensitivity but is restricted to a few specific, pre-defined pathway components. While this may be an appropriate strategy for detecting lowly expressed cytokine genes [11], it limits our understanding of the broader transcriptomic changes induced by poly(I:C) stimulation. Another limitation arises from the non-pathogenic nature of our immune stimulation. Although poly(I:C) treatment is convenient for experimental set-ups in basic research, it suffers from limited applicability of the research results on specific veterinary conditions [69–71]. Finally, owing to the lack of comparable data in other avian species, it remains unclear how universal the regulatory patterns observed in our study are. In conclusion, our study is, to our knowledge, the first to explore the *in vivo* immune response to poly(I:C) in parrots, and also to our knowledge, the first in birds to address the expression patterns of *NLRP3* and *CASP1* genes following poly(I:C) treatment in both the ileum and brain. It contributes to foundational knowledge on the regulation of avian immune defence, and its similarities and differences compared to humans and other mammals. The observed temporal dynamics and expression profiles of pro-inflammatory cytokines highlight the intense immune crosstalk between the periphery and CNS during viral challenges. Our results demonstrate that parrots are susceptible to severe neuroinflammation induced by peripheral immune activation. This is important for establishing parrots as a novel model in neuroimmunology, providing a basis for future interdisciplinary research, involving behavioural and veterinary studies. Future studies should broaden the scope of immune gene expression profiling in parrots by employing transcriptomics and investigating the long-term effects of poly(I:C) stimulation on immune responses.

## Data Availability

All data related to this article are included in the electronic supplementary material files [72].
